# Regulating the regulators via targeting CD38 in the tumor microenvironment

**DOI:** 10.3389/fimmu.2026.1745988

**Published:** 2026-02-03

**Authors:** Navnita Dutta, Nabanita Halder, Eduardo Nunes Chini, Sungjune Kim, Alak Manna

**Affiliations:** 1 Department of Cancer Biology, Mayo Clinic, Jacksonville, FL, United States; 2 Department of Neurosurgery, Mayo Clinic, Jacksonville, FL, United States; 3 Department of Anesthesiology, Mayo Clinic, Jacksonville, FL, United States; 4 Department of Radiation Oncology, Mayo Clinic, Jacksonville, FL, United States

**Keywords:** Breg, CD38, metabolism, NAD, tumor micro environment (TME)

## Abstract

The immunosuppressive tumor microenvironment (TME) remains a major barrier to effective cancer immunotherapy. Among the central regulators of immune suppression, CD38, a multifunctional ectoenzyme and surface glycoprotein, has emerged as a pivotal orchestrator. CD38 is abundantly expressed on regulatory T cells (Tregs), regulatory B cells (Bregs), myeloid-derived suppressor cells (MDSCs), tumor-associated macrophages (TAMs), and tumor-associated neutrophils (TANs), where it enhances survival, metabolic fitness, and suppressive activity. Invariant natural killer T (iNKT) cells, which can either promote or suppress antitumor immunity, also express CD38 upon activation, suggesting a role for CD38 in directing their context-dependent fate within the TME. Mechanistically, CD38 regulates immune suppression through NAD^+^ hydrolysis, calcium signaling, and promotion of fatty acid oxidation (FAO) while impairing effector T-cell glycolysis and mitochondrial fitness under chronic hypoxia—conditions that favor exhaustion rather than enhanced cytotoxicity. By depleting extracellular NAD^+^, CD38 diminishes glycolysis and mitochondrial oxidative phosphorylation in effector T cells, while sustaining regulatory cell persistence through FAO. Its enzymatic products, cyclic ADP-ribose (cADPR) and NAADP, further mobilize calcium fluxes that reinforce suppressive function. CD38 also integrates with hypoxia-driven pathways; in CD38^+^ Bregs, stabilization of HIF-1α and induction of FAO-related genes such as CPT1A and PPARα/γ promote angiogenesis, immune-evasion, and therapeutic resistance. Therapeutically, targeting CD38 with monoclonal-antibodies, small-molecule inhibitors, or combinations with checkpoint blockade and macrophage-reprogramming agents has shown promise. Such interventions reverse immune suppression, restore effector T cell activity, and enhance tumor responsiveness to immunotherapy. In summary, CD38 functions as both a metabolic regulator and an immunologic checkpoint, coordinating suppressive networks and shaping iNKT cell fate. These multifaceted roles position CD38 as a transformative target for next-generation immunotherapies.

## Highlights

- Regulating the Regulators: CD38 is not merely a marker or isolated checkpoint; it acts as a master regulator that sustains multiple immunosuppressive cell populations (Tregs, Bregs, MDSCs, TAMs, TANs, and regulatory NK cells) through shared metabolic and signaling pathways.
- CD38-driven NAD^+^ depletion, generation of second messengers (cADPR, NAADP), and promotion of FAO converge to maintain the survival, metabolic fitness, and suppressive function of regulatory immune cells within the TME.
- Beyond its ectoenzyme role, CD38 coordinates calcium signaling and transcriptional programs (STAT3, NFAT, NF-κB) that reinforce immunosuppressive phenotypes, positioning CD38 as a metabolic checkpoint and signaling orchestrator.
- Targeting CD38 offers a unique opportunity to dismantle interconnected suppressive circuits rather than addressing individual cell types in isolation. Strategies include monoclonal antibodies, bispecific constructs, antibody-drug conjugates, and rational combinations with checkpoint inhibitors or metabolic modulators.
- Emerging evidence on CD38’s intracellular localization (mitochondria, nucleus) and its role in epigenetic regulation and mitochondrial NAD^+^ pools expand its therapeutic relevance beyond surface depletion.
- High CD38 expression correlates with resistance to immune checkpoint blockade and poor prognosis in solid tumors. Selective CD38-targeting approaches could overcome these barriers and unlock the full potential of immunotherapy.

## 1 Introduction

The immunosuppressive TME is a hallmark of tumors and poses a significant challenge to effective anti-tumor immunity. This hostile TME undermines the immune system’s natural ability to recognize and eliminate cancer cells and substantially limits the success of modern immunotherapies, including immune checkpoint inhibitors, adoptive cell therapies, and cancer vaccines (1–3).

The TME is a dynamic ecosystem composed of tumor cells, stromal elements, and immune populations that collectively shape cancer progression and therapeutic response. While hypoxia, nutrient deprivation, and immunosuppressive cytokines such as IL-10 and TGF-β are well-recognized features, the focus of this review is on how these conditions converge on CD38-driven metabolic and signaling pathways. Rather than detailing all generic immune-evasion mechanisms, we emphasize CD38 as a central node that integrates NAD^+^ hydrolysis, Ca²^+^ signaling, and FAO to sustain regulatory immune subsets (namely, Tregs, Bregs, MDSCs, TAMs, and TANs) and blunt the effector immune cell function within the TME. What has been lacking is an integrated framework that defines CD38 as a master regulator of immunosuppressive networks within the tumor microenvironment (TME), a dynamic ecosystem integrating tumor cells, stromal and immune components, extracellular matrix, and soluble mediators (4–6).

This intricate composition of the TME establishes both physical and functional barriers that impede immune cell infiltration and their anti-tumor activity. CD38 emerges as a central orchestrator within this network, integrating metabolic and signaling pathways that sustain immunosuppressive circuits. Multiple mechanisms contribute to the immunosuppressive nature of the TME, enabling tumors to evade immune surveillance, resist immunotherapy, and promote recurrence and progression. **1)** CD38+regulatory immune cells including Tregs, Bregs, MSDCs, TAM and TANs suppress effector responses by releasing immunosuppressive cytokines (e.g., IL-10, TGF-β) and exerting contact-dependent or independent inhibition of T cell functions (7, 8). CD38 amplifies these effects by depleting NAD^+^, impairing glycolysis in effector T cells, and promoting FAO in regulatory subsets, thereby reinforcing their survival and suppressive function. MDSCs further suppress T cell and NK cell activation through metabolic disruption and production of arginase, nitric oxide (NO), and reactive oxygen species (ROS) (9) while CD38-driven calcium signaling activates STAT3 and NF-κB pathways that sustain these phenotypes. M2-polarized TAMs (also express high CD38) promote tumor progression by secreting anti-inflammatory cytokines (e.g. IL-6, IL-4, IL10 and TGFβ) and supporting tissue remodeling (10). **2)** Upregulation of immune checkpoints, such as PD-L1, MHC class II, HMGB1, CD152 expression on tumor and stromal cells often associated with high CD38 expressionbinds to PD-1, LAG3, TIM3 and TIGIT on T cells respectively inducing T cell exhaustion and dysfunction (11, 12). **3)** Metabolic limitations, including hypoxia and glucose deprivation, converge with CD38-mediated NAD^+^ depletion and adenosine accumulation to impair cytotoxic T cell fitness (13–15) and **4)** The extra cellular matrix (ECM) and abnormal vasculature, further restrict immune cell trafficking and penetration into the tumor core (16–18). To overcome these challenges, emerging strategies are focusing on targeting CD38 to dismantle interconnected suppressive circuits (19–22). Approaches include monoclonal antibodies (e.g., Daratumumab, Isatuximab), bispecific constructs (23), and rational combinations with checkpoint inhibitors, metabolic modulators (FK866) (24), or TAM-reprogramming agents. A deeper understanding of CD38-driven immune-metabolic control within the TME is essential for developing next-generation treatments that restore immune competence and improve patient outcomes.

Our review introduces the conceptual framework of “regulating the regulators” by targeting CD38. Rather than viewing CD38 as a marker or isolated checkpoint, we synthesize evidence demonstrating how CD38-driven mechanisms—including NAD^+^ depletion, generation of second messengers (cADPR, NAADP), and promotion of FAO-converge to sustain the survival, metabolic fitness, and suppressive function of diverse immunoregulatory subsets within the tumor microenvironment (TME). These includes Tregs (25), MDSCs (26, 27), Bregs (28), M2-TAMs (29, 30), TANs (31, 32), and regulatory NK cells (33) as well as tumor cells themselves which frequently express CD38 on their surface. The CD38, a multifunctional ectoenzyme and cell receptor (34–36), plays a central role in tumor immune evasion, metabolic adaptation, favoring the establishment of an immunosuppressive milieu (31, 35, 37). By coordinating shared metabolic and signaling pathways across multiple suppressive cell populations, CD38 functions as a strategic node for ‘regulating the regulators’ within the TME.

Mechanistically CD38 contributes to immune escape through activation of a noncanonical adenosinergic pathway, catalyzing the conversion of NAD^+^ to ADP-ribose and (38, 39) and ultimately to adenosine, via cooperation with CD203a and CD73 (40). Adenosine then acts on A2A and A2B receptors to inhibit effector T cell and NK cell functions (41). CD38^+^ Tregs (CD4^+^CD25^+^FoxP3^+^CD38^+^) exhibit enhanced suppressive capacity and are enriched in the TME, secreting IL-10 and TGF-β to reinforce cytotoxic T cell (CTL) dysfunction (42, 43). Similarly CD38^+^MDSCs maintain potent immunosuppressive activity, metabolic adaptation, and tumor-promoting interactions (27); while CD38^+^ TAMs adopt an M2-like phenotype that supports tumor growth, matrix remodeling, and immune suppression (29); High CD38 expression on CD8^+^/CD4^+^ T cells correlates with exhaustion markers (PD-1, LAG-3) and poor response to checkpoint blockade (44) and CD38 activity is further linked to IFN-γ-induced expression in tumor and immune cells, driving resistance mechanisms (45, 46); CD38+ TANs enriched in N2-like subtypes, promote PD-1, TIM-3, and LAG-3 expression on neighboring T cells via adenosine signaling and secrete IL-10, TGF-β, and ROS (47, 48), while also producing VEGF, MMP9, and IL-8 to facilitate angiogenesis, ECM remodeling, and metastasis (30, 49). Finally, CD38^+^ regulatory NK cells adopt pro-tumor functions, suppressing T cells through inhibitory cytokines and contributing to an adenosine-rich, metabolically suppressive environment (19, 40). Collectively, these findings underscore CD38 as a master regulator of immunosuppressive networks and a compelling therapeutic target for dismantling the interconnected circuits that sustain tumor immune evasion.

The immune suppressive nature of CD38+ immune cells also contributes significantly to resistance against immune checkpoint therapy, as high CD38 expression is strongly associated with resistance to PD-1/PD-L1 blockade by promoting immunosuppression and limiting T cell infiltration and function (40). Anti-CD38 monoclonal antibodies (e.g., Daratumumab, Isatuximab) already approved for multiple myeloma, are being explored in solid tumors (50, 51). These agents deplete CD38^+^ immunosuppressive cells and remodel the TME toward a more immunostimulatory state. Combination strategies—such as pairing anti-CD38 therapy with PD-1/PD-L1 (52, 53) or CTLA-4 blockade (54), A2A receptor antagonists and adaptive cell therapies including CAR-T or NK cell approaches (55) shows synergistic effects. Targeting CD38 not only reduces immunosuppressive cytokines, but also enhances effector T cell infiltration, and antigen presentation (56).

**Figure 1:**
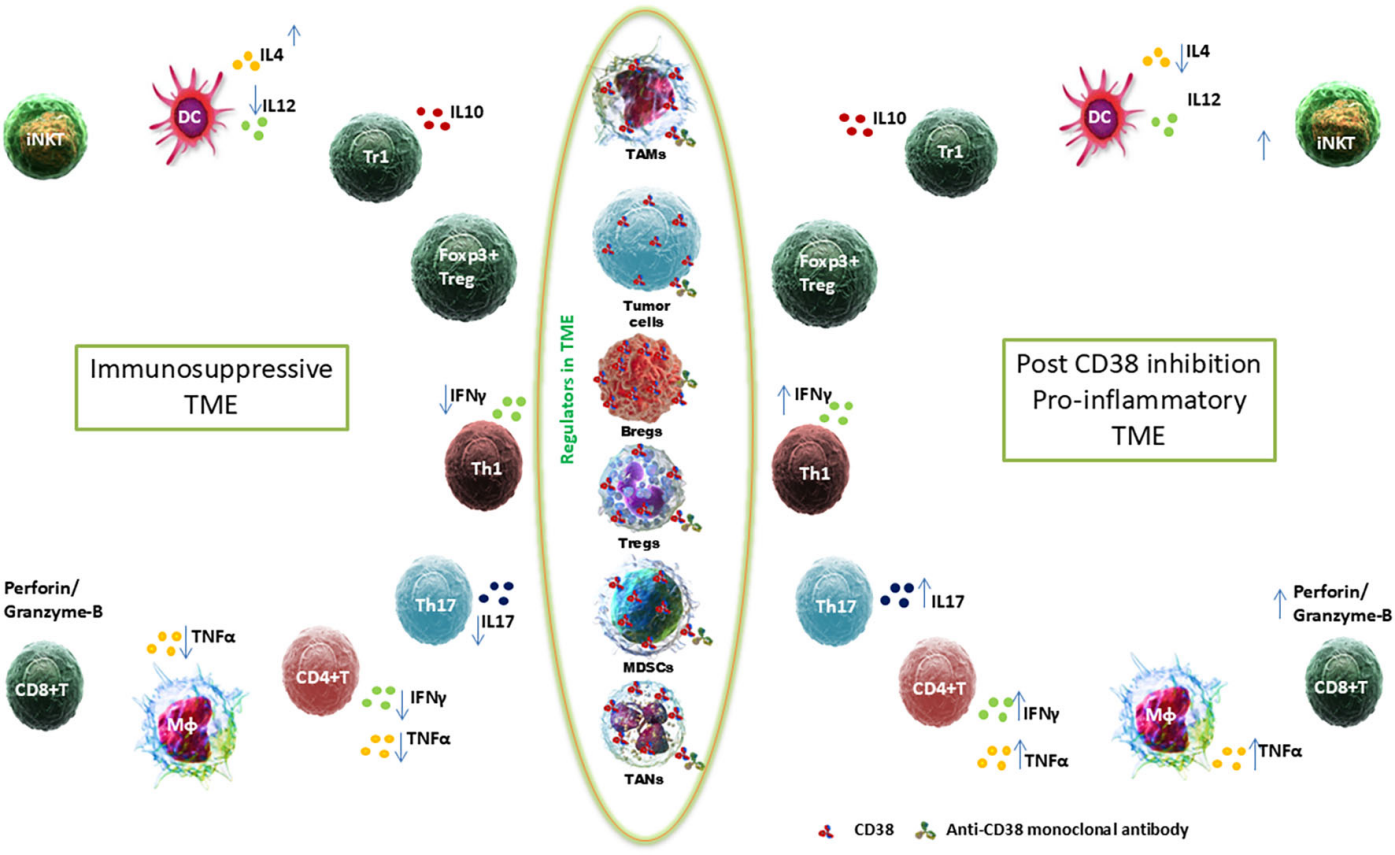
Shifting Immunoregulatory Balance in the TME via CD38 Inhibition. The left panel depicts an immunosuppressive TME characterized by the presence of TAMs, Bregs, Tregs, MDSCs, and TANs. Immune cells such as dendritic cells (DCs), invariant natural killer T (iNKT) cells, Tr1 cells, and Foxp3^+^ Tregs are shown producing immunosuppressive cytokines including IL-10 and IL-4. The right panel represents a pro-inflammatory TME post-CD38 inhibition, marked by enhanced activity of Th1 and Th17 cells, CD4^+^ and CD8^+^ T cells, and macrophages (Mφ), producing cytokines and cytotoxic molecules such as IFNγ, TNFα, IL-17, perforin, and granzyme B. The central column highlights the reduction of immunosuppressive components in the TME due to CD38 blockade. CD38 molecules are indicated by red triangles, and anti-CD38 monoclonal antibodies are represented by green Y-shaped symbols.

Therefore, regulating immunosuppressive and immune regulatory cells through CD38 inhibition represents a promising approach in cancer immunotherapy. By dismantling multiple suppressive mechanisms within the TME modulating cytokine networks, reducing the abundance of CD38^+^ regulatory cells, and reversing immune exhaustion, CD38 targeted strategies can reshape the immune landscape toward an anti-tumor phenotype. Integrating CD38 blockade with checkpoint inhibitors and metabolic reprogramming agents holds strong translational potential for overcoming resistance and improving clinical outcomes (**Figure 1**).

## 2 Immune escape of tumor cells by imposing immune editing in tumor micro-environment

Tumor cells evolve mechanisms to evade immune surveillance through a dynamic process known as cancer immune editing (57), which ultimately leads to reduced immunogenicity and immune escape. This process occurs in three distinct phases: *elimination, equilibrium*, and *escape*. In the elimination phase, immune cells recognize and destroy highly immunogenic tumor cells. However, under selective pressure, tumor variants with immune-resistant features survive and persist in a state of equilibrium. Eventually, during the escape phase, these tumor cells acquire additional adaptations that allow them to evade immune detection and destruction-including upregulation of CD38 (58, 59). CD38 expression on tumor and infiltrating regulatory cells amplifies immune suppression by hydrolyzing NAD^+^ and generating adenosine, which impairs effector T-cell metabolism and promotes exhaustion. Concurrently, CD38-driven calcium signaling and metabolic reprogramming sustain Tregs, Bregs, MDSCs, and TAMs, reinforcing immunosuppressive networks within the tumor microenvironment (55–57). Tumor cells employ multiple immune escape mechanisms, and CD38 acts as a critical metabolic and signaling checkpoint within these strategies. Key immune evasion strategies include: **1)** Downregulation or loss of antigen presentation machinery, such as MHC class I, to avoid recognition by cytotoxic T lymphocytes (60, 61), **2)** Expression of “don’t-eat-me” signals, such as CD47, which inhibit phagocytosis by antigen-presenting cells (APCs) like macrophages (62)- tumors exploit CD38 to reinforce immunosuppression. CD38 enzymatically hydrolyzes NAD^+^, generating adenosine that activates A2A receptors on effector T cells, suppressing glycolysis and mitochondrial OXPHOS, and promoting exhaustion; **3)** Secretion of immunosuppressive cytokines, including TGF-β and IL-10, along with recruitment of immunoregulatory cells such as Tregs, MDSCs, TAMs, and Bregs, which collectively suppress effector T cell and NK cell function (59); and **4)**.

These CD38-driven processes complement physical and biochemical remodeling of the tumor microenvironment (TME), creating barriers that limit immune infiltration and activity (63, 64).

## 3 Tumor cells educate and reprogram immune cells in TME

The immune cells within the TME play essential roles in promoting tumor immune evasion and disease progression. Among these, CD38 expression is increasingly recognized as a defining feature of multiple immunosuppressive subsets. Key CD38+immunosuppressive cellular components in this landscape include Tregs (63, 65), Bregs (66, 67), NK cells (68, 69), MDSCs (70, 71), M2-polarized TAMs (72, 73), and TANs (74, 75). Tumor cells actively reprogram these immune populations through a sophisticated regulatory network, and CD38 serves as a critical metabolic checkpoint within this network. Beyond classical immune regulatory mechanisms, tumor cell **1)** Downregulate MHC class I molecules, allowing them to escape recognition by cytotoxic T lymphocytes (CTLs) (60, 76). **2)** Release of soluble inhibitory ligands that suppress activating immune receptors: MICA/B shedding, which disrupts NK cell activation via the NKG2D receptor (77, 78); Galectin-3 and BAG6/BAT3, which inhibit NKp30-mediated signaling in NK cells (79); PD-L1 expression, engaging PD-1 on T cells to dampen their activation (80, 81)-tumors exploit CD38 to reinforce immune suppression. **3)** Impairment of immune cell recruitment by disrupting tumor vasculature and altering chemokine gradients, thus hindering effective trafficking of antigen-presenting cells (APCs) and effector T cells. CD38 contributes to this barrier by promoting adenosine accumulation and hypoxia-driven signaling. Adenosine receptor activation reduces endothelial adhesion molecule expression and alters chemokine gradients, further limiting immune cell infiltration (82, 83). **4)** Chemokine-driven recruitment and polarization of CD38+immunosuppressive cells (84): MDSCs are recruited via CXCL5, CXCL8, and CXCL12 (85, 86)Tregs are attracted by CCL3, CCL4, and CCL5 (87, 88)Macrophages are polarized from an M1 (anti-tumor) to M2 (pro-tumor) phenotype in response to tumor-derived cytokines and environmental signals (89, 90). Importantly, many of these recruited populations express **CD38**, which amplifies their suppressive capacity through NAD^+^ hydrolysis, adenosinergic signaling, and calcium-mediated transcriptional activation. **5)** Tumor cells and CD38^+^ regulatory immune subsets secrete immunosuppressive cytokines, including IL-10, IL-35, and TGF-β, by tumor cells and regulatory immune cells, which inhibits T cell effector functions and suppresses dendritic cell (DC) maturation (91, 92). **6)** Upregulation of immune checkpoint molecules, particularly PD-L1, which binds PD-1 on T cells and triggers intracellular signaling that suppresses T cell activation. This includes dephosphorylation of RAS and PI3K, leading to inhibition of downstream AKT and ERK pathways critical for T cell survival and function (93, 94). Thus CD38-driven metabolic stress and checkpoint signaling create a synergistic barrier to effective anti-tumor immunity. **7)** Metabolic reprogramming of the TME, including a shift toward aerobic glycolysis (Warburg effect), enables tumor cells to outcompete immune cells for nutrients such as glucose and amino acids, thereby impairing effector T cell metabolism and contributing to immune escape (95, 96). Beyond this classical mechanism, CD38 amplifies metabolic suppression by hydrolyzing NAD^+^ and generating adenosine, which inhibits glycolysis and OXPHOS in effector T cells. Simultaneously, CD38^+^ regulatory populations adapt through FAO and calcium-dependent signaling, sustaining their survival under nutrient-limited and hypoxic conditions. This CD38-driven metabolic asymmetry reinforces immune suppression and resistance to immunotherapy (93, 94).

## 4 The immune suppressive cells in the TME contribute to immune escape via secreting cytokines

Those immune suppressive cells in the TME contribute to immune escape primarily through the secretion of immunosuppressive cytokines, notably interleukin-10 (IL-10), transforming growth factor-beta (TGF-β), and interleukin-35 (IL-35) (11, 91, 92). These cytokines create a tolerogenic microenvironment that suppresses effective anti-tumor and anti-pathogen immunity. IL-10 inhibits antigen-presenting cells by downregulating MHC class II and costimulatory molecules (CD80, CD86, CD40) on dendritic cells and macrophages (97). It also suppresses the production of pro-inflammatory cytokines such as IL-12, TNF-α, and IFN-γ, leading to a dampened Th1 response (98, 99). Additionally, IL-10 directly inhibits the activity of CD4 effector T cells and natural killer (NK) cells (100). **TGF-β** promotes immune tolerance by inducing the differentiation of naïve CD4^+^ T cells into regulatory T cells (Tregs) (101). It also inhibits the cytotoxic function and proliferation of CD8^+^ T cells as well as NK cells and downregulates the expression of co-stimulatory molecules on APCs, further impairing effective antigen presentation (102). **IL-35** suppresses immune responses by inhibiting T cell proliferation, blocking effector T cell activation, and promoting T cell anergy (103, 104). It also facilitates the expansion of IL-35-producing regulatory T and B cells, reinforcing a suppressive immunological network (105). Taken together, these cytokines enable Bregs to orchestrate a highly immunosuppressive environment, facilitating immune evasion in the context of cancer, chronic infections, and other immunopathological conditions.

Phenotypically, a substantial fraction of immunosuppressive and regulatory cells within the TME express CD38 and produce IL-10, underscoring their role in immune tolerance. Specific marker profiles include: *Tregs:* CD4^+^ CD25^+^ FoxP3^+^ CD38^+^ IL-10^+^ (106, 107); *Bregs:* CD19^+^/CD20^+^ CD24^hi CD38^hi IL-10^+^ (108, 109); *Monocytic MDSCs (M-MDSCs):* CD11b^+^ CD14^+^ HLA-DR^low/- CD15^-^ CD38^+^ IL-10^+^ (26, 110, 111); *Polymorphonuclear MDSCs (PMN-MDSCs):* CD11b^+^ CD14^-^ CD15^+^ or CD66b^+^ CD38^+^ IL-10^+^ (26, 111, 112); *TAMs:* CD11b^+^ CD68^+^ CD163^+^ CD206^+^ CD38^+^ IL-10^+^ (29, 113, 114); *TANs:* CD11b^+^ CD66b^+^ CD16^+^ CD38^+^ IL-10^+^ (31, 115–117) and *Regulatory natural killer (NK-Reg) cells:* CD56^bright CD16^-^ NKG2D^+^ TIGIT^+^ CD38^+^ IL-10^+^ (31, 64, 92, 118–120).

These CD38+cells mutually reinforce immune tolerance through crosstalk and cooperative regulation of naïve immune cells. Collectively, Tregs, MDSCs, TAMs, TANs, regulatory NK cells, and Bregs suppress the activity of CD8^+^ T cells, Th1 cells, and cytotoxic NK cells, primarily by producing immunosuppressive cytokines such as IL-10 and TGF-β. They also promote recruitment, differentiation, and expansion of additional regulatory populations, establishing a feedback loop that sustains an immunosuppressive microenvironment (92, 121). Importantly, this coordinated immune regulation contributes to resistance against immune checkpoint blockade (ICB) therapies, including anti-PD-1, anti-CTLA-4, and anti-TIGIT treatments (122–124). **Table 1**, summarizes the major immunosuppressive cell populations within the TME—Tregs, MDSCs, TAMs, TANs, regulatory NK cells, and Bregs—and describes how each population supports other suppressive cells through cytokine secretion, metabolic inhibition, and cross−regulatory interactions. It also outlines the immune checkpoint molecules expressed by each cell type, including PD−1/PD−L1, CTLA−4, TIM−3, TIGIT, VISTA, CD38, and Galectin−9, and highlights their roles in maintaining T−cell dysfunction, promoting Treg and myeloid suppressor expansion, and sustaining immunosuppression even in the context of immune checkpoint blockade. Together, these mechanisms illustrate the coordinated network of suppressive pathways that reinforces resistance to antitumor immunity and limits the efficacy of checkpoint inhibitors.

**Table 1:** Immunosuppressive roles of key immune cell subsets in the TME and their checkpoint-related mechanisms.

Immune cells	How they support others in TME	Checkpoint-related mechanisms
Tregs (CD4^+^FoxP3^+^)	-Secrete IL-10 and TGF-β to inhibit effector T cells, DCs, NK cells, and promote MDSC and TAM expansion (121, 125) -Express CTLA-4, which downregulates costimulatory molecules on DCs and enhances MDSC suppressive functions (126, 127)	-Express CTLA-4, PD-1, TIGIT -Suppress CD8^+^ T cells even during anti-PD-1 therapy (128, 129) - Enhance PD-L1 expression on APCs and MDSCs (130–132)
MDSCs	- Produce IL-10, Arg1, and ROS to suppress T and NK cells (133, 134) - Induce Treg differentiation via TGF-β, IL-10, and IDO (135–137) - Recruit TAMs and polarize them to M2 phenotype (90, 138, 139)	- Express PD-L1, CD38, VISTA, Gal-9 (140–142) - Expand Tregs in response to checkpoint blockade (143, 144) - Block CD8^+^ T cell infiltration and function (145–147)
TAMs	- Secrete IL-10, TGF-β, VEGF, and CCL22 to attract Tregs (139, 148, 149) - Enhance MDSC function and survival via cytokines and metabolic remodeling (150) - Suppress NK cells via IL-10 and arginase (138, 148)	- Express high PD-L1, B7-H4, VISTA, CD38 (29, 151–155); - Secrete IL-10 and TGF-β to maintain T cell exhaustion (11, 156) - Suppress responses even after anti-PD-1/PD-L1 therapy (157)
TANs	- Release IL-10, ROS, and MMPs that suppress T cells and support TAM and MDSC function (115, 158–160) - Can convert into MDSC-like or macrophage-like cells under certain conditions (161, 162)	- Express PD-L1 and produce ROS (115, 163, 164) - May suppress CD8^+^ T cells independently of PD-1 (165)
Regulatory NK cells	- Produce IL-10 to suppress T cells and DCs (166, 167) - modulate Treg expansion (168); 160, 161 - Reduced cytotoxicity enables TAMs and MDSCs to accumulate unchallenged (169)	- Upregulate PD-1, TIM-3, TIGIT (170) - Less responsive to checkpoint blockade (171) - Can modulate Treg expansion/functions via IL-10 (172, 173)
Bregs	- Promote Treg induction, - suppress CD8^+^ T cells and DCs, - support MDSCs and TAMs function (174), - secrete IL-10, TGF-β, and IL-35 (175, 176)	- Induce Tregs, express PD-L1, IL-10, Galectin-9 (175, 177) - Promote checkpoint ligand expression on other immune cells (178, 179) - Interfere with CD8^+^ T cell activation directly and indirectly (180)

## 5 The immune suppressive immune cells in the TME contribute to metabolic reprogramming

Immunosuppressive regulatory cells in the TME drive metabolic reprogramming that promotes tumor progression and immune tolerance. They compete for nutrients, produce suppressive metabolites, and adapt their metabolism to survive, inhibit immune responses, and foster therapeutic resistance. Key populations—MDSCs, TAMs, and Tregs—consume high levels of glucose, amino acids (e.g., L-arginine, tryptophan), and lipids, reshaping the metabolic landscape to favor tumor survival (181, 182), thereby depriving effector T cells and natural killer (NK) cells of essential energy substrates. Arginase-1, predominantly expressed by MDSCs and TAMs, deplete L-arginine, a critical nutrient for T cell proliferation and function. Similarly, the indoleamine 2,3-dioxygenase (IDO) enzyme catabolizes tryptophan into immunosuppressive metabolites like kynurenine, which further suppress T cell activity and promote regulatory phenotypes (183–185).

These regulatory cells also facilitate the accumulation of adenosine, a potent immunosuppressive metabolite, by upregulating ectonucleotides including CD39, CD203a (ENPP1), and CD73. Accumulated adenosine binds to A2A receptors on Tregs and MDSCs, activating AMP-activated protein kinase (AMPK) and shifting cellular metabolism toward mitochondrial FAO, thereby reinforcing their suppressive functions (186, 187), while suppressing the TCR signaling and reducing glycolytic flux, thereby impairing mitochondrial OXPHOS and promoting exhaustion. Additionally, the accumulation of lactate contributes to the acidification of the TME, impairing effector T cell function and enhancing Treg stability. Regulatory cells also produce elevated levels of reactive oxygen species (ROS) and nitric oxide (NO), which induce oxidative stress and further suppress immune responses (188, 189). Preferential utilization of FAO and oxidative phosphorylation supports the energy demands and suppressive phenotypes of these cells. Moreover, they help maintain hypoxic conditions within the TME, activating hypoxia-inducible factor-1α (HIF-1α) and inducing the expression of FAO-related genes such as CPT1A, PPARα, and PPARγ, which further reinforce immunosuppressive metabolic programming (176).

## 6 Role of CD38 in immune regulatory cell in TME

CD38 plays a critical immunosuppressive role in immune regulatory cells within the TME. These regulatory cells, including Tregs (25, 190), MDSCs (26, 27), and TAMs (30), contribute to tumor immune evasion. Below is a focused explanation of CD38’s role in immune regulatory cells in the TME. CD38 is a multifunctional ectoenzyme and type II transmembrane glycoprotein, primarily known for its NADase activity, with catalytic domains facing the extracellular space (35, 38, 191). However, it is also localized to intracellular compartments, including mitochondria (the outer mitochondrial membrane, inner mitochondrial membrane, mitochondria-associated membranes (MAMs) where it maintains mitochondrial NAD^+^ pools, impacting oxidative metabolism, apoptosis, and redox balance) and the nucleus (nuclear CD38 hydrolyzes NAD^+^, affecting SIRT1 and PARP activity, thus linking it to epigenetic regulation, DNA repair, and transcriptional control), indicating broader regulatory roles in metabolism (192–195), signaling (40), and epigenetic control (196). The key enzymatic functions of CD38 are 1) Hydrolysis of NAD^+^ and NMN: CD38 catalyzes the hydrolysis of nicotinamide adenine dinucleotide (NAD^+^) and nicotinamide mononucleotide (NMN) to ADP-ribose (ADPR) and nicotinamide (197–199), thereby regulating the availability of NAD^+^—a critical cofactor for a) T cell metabolism and function; b) Sirtuins (SIRT1–SIRT7) involved in post-translational modifications and c) Poly(ADP-ribose) polymerases (PARPs) essential for DNA repair. Regulatory populations (Tregs, MDSCs, TAMs) adapt to NAD^+^ scarcity and hypoxia by upregulating FAO and stress-responsive pathways such as the **SIRT1/NLRP3 axis**, which promotes survival under nutrient-limited conditions (200). 2) Cyclization of NAD^+^ to cyclic ADP-ribose (cADPR): CD38 converts NAD^+^ into cADPR, a potent intracellular second messenger that mobilizes Ca²^+^ from intracellular stores, particularly via ryanodine receptors and regulates T cell activation, dendritic cell maturation, and immune synapse formation (201). 3) Hydrolysis of cADPR to ADPR: This step controls the amplitude and duration of cADPR-mediated Ca²^+^ signaling, fine-tuning immune responses and intracellular communication (202, 203). 4) Conversion of NADP^+^ to NAADP: CD38 can also metabolize NADP^+^ to generate nicotinic acid adenine dinucleotide phosphate (NAADP), one of the most potent known Ca²^+^-mobilizing agents (204–207). NAADP regulates Lysosomal Ca²^+^ signaling, Autophagy and Cell survival and trafficking (208, 209). CD38 thus creates a metabolic asymmetry: effector cells become exhausted due to impaired glycolysis/OXPHOS, while regulatory cells thrive via FAO and calcium-driven signaling, sustaining immunosuppression in the TME.

### 6.1 The expression of CD38 on Bregs and functional implication in the TME

**Figure 2:**
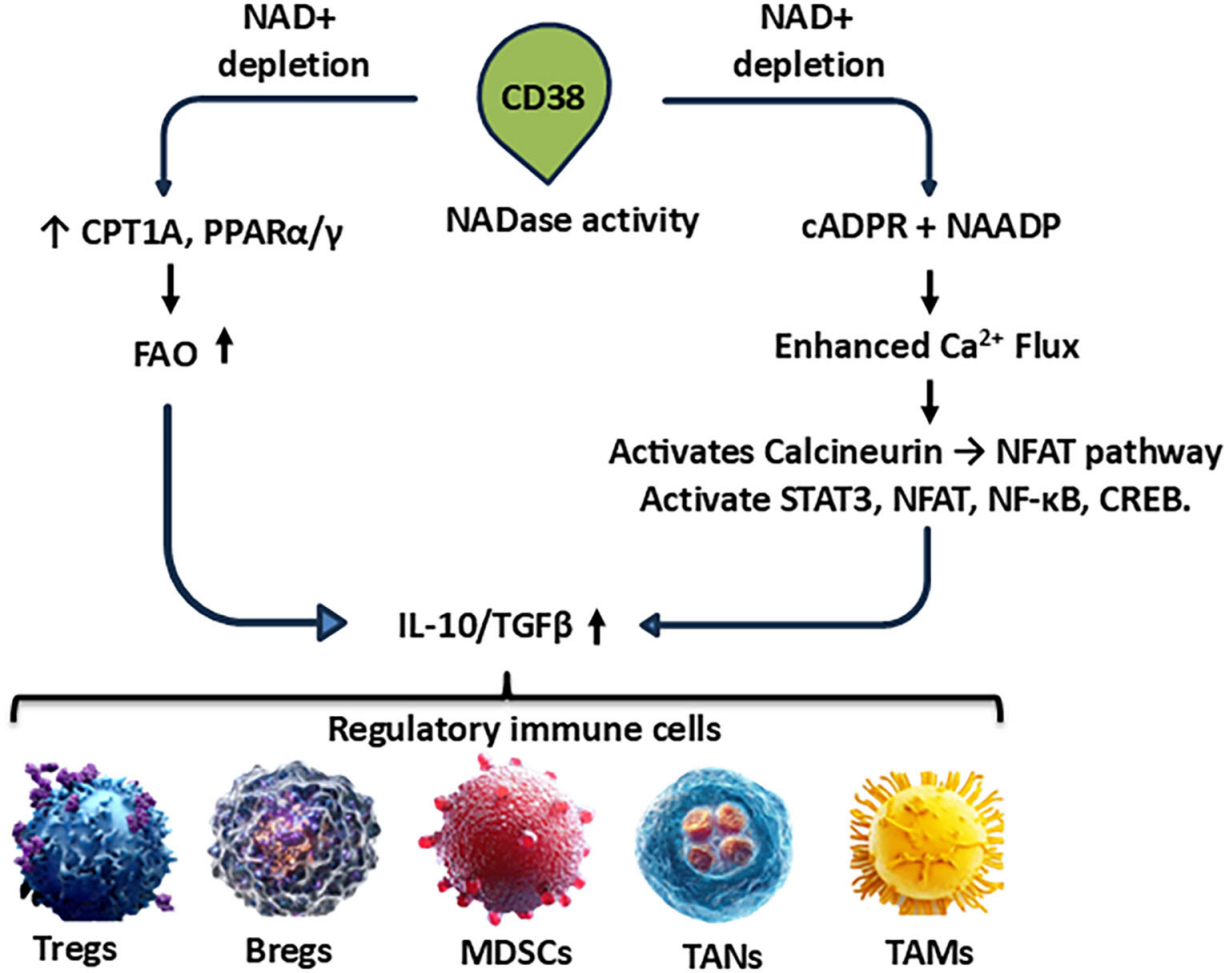
CD38^+^ Bregs orchestrate metabolic suppression in the TME. Regulatory B cells expressing CD38 degrade extracellular NAD^+^ into ADP-ribose and adenosine. Adenosine activates A2A receptors on effector T cells, triggering cAMP accumulation and downstream signaling that inhibits T cell proliferation and cytotoxicity. Concurrently, Bregs secrete IL-10 and TGF-β, promoting Treg differentiation and reinforcing immune suppression. Under chronic hypoxic conditions, effector T-cell glycolysis is impaired and FAO is increased. CD38 inhibitors can block NAD^+^ catabolism and restore T cell metabolic function.

CD38 plays a pivotal role in B cell biology, particularly in the development, activation, and immunoregulatory function of regulatory B cells (Bregs, **Figure 2**) (210, 211). Upon engagement, CD38 functions as both a signaling molecule and an enzyme, contributing to the immunomodulatory capacity of Bregs via enhances B cell receptor (BCR) signaling (212). Thereby it is amplifying antigen-specific activation, promoting antigen processing and presentation, facilitating interaction with T cells (213, 214). CD38 upregulates co-stimulatory molecules such as CD80 and CD86, supporting the immune-regulatory dialogue between Bregs and effector T cells (215, 216). Through these actions, CD38 contributes to the maintenance of peripheral tolerance, and helps Bregs execute their suppressive functions, primarily via cytokines such as IL-10 and TGF-β (215). These effects are particularly relevant in inflammatory settings, including the TME, where Bregs suppress anti-tumor immune responses and contribute to immune evasion.

#### 6.1.1 Calcium signaling and transcription factors

Following CD38 activation, the enzymatic production of cyclic ADP-ribose (cADPR) and NAADP from NAD+ mobilizes intracellular Ca²^+^ stores via ryanodine receptors and endo-lysosomal channels, respectively (205, 217–219). The resulting elevation in cytosolic Ca²^+^ serves as a central second messenger that activates a network of transcription factors (STAT3, Blimp1, IRF4, c-Maf, AhR, Bcl-6, NF-kB and HIF-1a) essential for the expression of IL-10 and the maintenance of Breg identity (178, 220). Among the most critical of these transcription factors are STAT3, and cMaf which becomes activated through both Ca²^+^/calcineurin/NFAT and cytokine receptor engagement, particularly IL-6 and IL-21 receptors (221, 222) whereas BATF and IRF4 activation is more dependent on BCR strength and AP-1 cooperation during effector differentiation (223) similar like T cells. Once phosphorylated (typically via JAK kinases), STAT3 dimerizes and translocate to the nucleus, where it binds to the IL10 promoter, initiating transcription (224, 225). STAT3 activation is tightly linked to Breg function, as it supports IL-10 production, cell survival, and resistance to pro-inflammatory cues within the TME (226). NFAT is a Ca²^+^-sensitive transcription factor activated downstream of calcineurin, a phosphatase that is triggered by elevated Ca²^+^. Once dephosphorylated, NFAT translocates to the nucleus and cooperates with other transcription factors such as AP-1 and STAT3 to drive IL-10 gene expression (227, 228). In Bregs, NFAT not only contributes to IL-10 transcription, but also modulates expression of surface molecules like CD80 and CD86 and may influence B cell tolerance mechanisms (222). CREB (cAMP Response Element-Binding Protein) is another transcription factor activated by Ca²^+^-calmodulin-dependent kinases (CaMKs) and protein kinase A (PKA) pathways (229). Upon phosphorylation, CREB binds to cAMP response elements (CREs) in the IL10 promoter and enhances transcription. CREB is especially important for the sustained expression of IL-10 under chronic stimulation and works synergistically with NFAT and STAT3 (230, 231). These transcription factors do not act in isolation. Instead, they form a complex regulatory network and integration of these signals ensures that Bregs maintain a robust immunosuppressive phenotype, particularly in inflammatory or tumorigenic environments, where immune regulation is critical to tissue homeostasis and tumor immune evasion.

#### 6.1.2 Signaling

In addition to its enzymatic functions, CD38 also acts as a co-stimulatory molecule, and its ligation triggers multiple intracellular signaling cascades critical for Breg identity and stability. PI3K/Akt pathway promotes cell survival, proliferation, and metabolic fitness, ensuring Breg resilience in stressful microenvironments, MAPK/ERK pathway contributes to IL-10 gene expression by modulating transcription factors and chromatin accessibility and NF-κB signaling regulates the transcription of immunosuppressive cytokines including IL-10 and TGF-β, reinforcing the suppressive phenotype of Bregs (232, 233). These interconnected signaling pathways not only facilitate cytokine production but also enhance resistance to apoptosis, support long-term immunosuppressive function and Enable Bregs to remain functionally active within inflammatory or tumorigenic environments, such as the TME (234, 235). By coordinating metabolic, survival, and cytokine signaling, CD38 equips Bregs with a robust toolkit to suppress effector immune responses, thus contributing to immune evasion and tumor progression in cancer settings.

#### 6.1.3 Metabolism

CD38^+^ regulatory B cells (Bregs) exert profound immuno-metabolic control within the TME by modulating both their own metabolism and that of surrounding immune and tumor cells via metabolic rewiring. Through their NADase activity, CD38^+^ Bregs deplete extracellular NAD^+^, a crucial cofactor for glycolysis and mitochondrial oxidative phosphorylation (OXPHOS) (42, 236–238). This NAD^+^ depletion impairs energy metabolism in neighboring immune cells, disrupting glycolysis and mitochondrial respiration, and forcing a metabolic shift toward FAO as an alternative energy source (239–241). This shift is not confined to Bregs alone, however, CD38^+^ Breg influences the metabolic programming of regulatory T cells (Tregs) and myeloid-derived suppressor cells (MDSCs), promoting a tolerogenic, FAO-driven metabolic phenotype (133). This adaptation enhances the longevity, suppressive capacity, and survival of these regulatory populations, establishing a metabolically immunosuppressive niche in the TME (236). Through secretion of interleukin-10 (IL-10) and transforming growth factor-β (TGF-β), Bregs also contributed to the maintenance of hypoxic conditions, promoting the stabilization and activation of hypoxia-inducible factor-1α (HIF-1α) in T cells. Importantly, transient HIF-1α activity during early activation supports glycolysis and effector differentiation, whereas chronic hypoxia and sustained HIF-1α signaling drive T-cell exhaustion (222, 242). Under these conditions, metabolic reprogramming favors regulatory phenotypes: upregulation of metabolic genes involved in FAO and hypoxic adaptation, such as carnitine palmitoyl transferase 1A (CPT1A) and peroxisome proliferator-activated receptor alpha (PPARα), occurs primarily in regulatory and memory subsets, not effector T cells (243–245). This adaptation, reinforced by CD38-driven NAD^+^ depletion and calcium signaling, sustains immunosuppressive cell survival and function while promoting tumor growth, angiogenesis, and immune evasion. Enhanced glycolysis and angiogenesis in tumor cells are promoted, supporting tumor growth, invasion, and vascular remodeling. Concurrently, the metabolic competition and accumulation of immunosuppressive metabolites suppress the metabolic fitness of cytotoxic T lymphocytes (CTLs), thereby impairing their effector functions and contributing to immune evasion (246–248). and polarization of macrophages toward the M2 phenotype, characterized by tissue remodeling and anti-inflammatory functions (249). Collectively, these mechanisms allow CD38^+^ Bregs to orchestrate a metabolically conditioned, immunosuppressive TME, which favors regulatory and tolerogenic immune cells while suppressing effector functions. This not only facilitates tumor progression, but also contributes to resistance to immunotherapy, making Breg-targeting strategies a compelling avenue for therapeutic intervention (25).

The dominance of CD38+IL10+regulatory cells within the TME has significant implications for the efficacy of immunotherapies. One of the most significant consequences of this immunosuppressive activity is the dampening of immune checkpoint blockade (ICB) efficacy, including anti-PD-1/PD-L1 and anti-CTLA-4 therapies (31, 40). By secreting immunosuppressive cytokines such as IL-10 and TGF-β, and through metabolic modulation of the TME, Bregs inhibit cytotoxic T lymphocyte (CTL) activation, proliferation, and survival—key processes required for effective ICB-mediated tumor clearance (92, 175, 250). This challenge is particularly pronounced in solid tumors, where Bregs are often enriched and contribute to a physically and metabolically restrictive TME. In such settings, the suppressive landscape also limits the efficacy of chimeric antigen receptor T cell (CAR-T) therapies by reducing T cell infiltration, persistence, and cytotoxic function (67, 251, 252). As a result, Breg-mediated suppression poses a critical barrier to both endogenous and adoptive immune responses, exacerbating resistance to immunotherapy. To address this resistance, several therapeutic strategies are under active investigation. 1) Cytokine Pathway Blockade: targeting the IL-10 and TGF-β signaling axes can alleviate Breg-induced immunosuppression including Neutralizing antibodies against IL-10 or TGF-β, small-molecule inhibitors of downstream signaling (e.g., STAT3, TGFβR), receptor antagonists block cytokine binding and downstream transcriptional effects. The overall aim is to restore CTL functionality, promote pro-inflammatory reprogramming of the TME, and improve responsiveness to checkpoint inhibitors (253–255). 2) Metabolic reprogramming: given the reliance of Bregs, Tregs, and MDSCs on FAO for energy and function, metabolic targeting offers a complementary strategy. Inhibitors of FAO pathways (e.g., CPT1A inhibitors) (248, 256–258) or agents that disrupt lipid uptake and processing can reduce the metabolic fitness of suppressive cells and tilt the TME toward immune-stimulation (259, 260). Though a certain degree of limitations to target FAO as memory T cells also rely on it. These interventions may promote effector T cell activity, limit the suppressive capacity of regulatory populations and synergize with ICB and CAR-T therapies in metabolically hostile tumors. Overcoming Breg-mediated resistance is a key frontier in improving cancer immunotherapy outcomes, especially in checkpoint-refractory tumors and solid malignancies. By combining cytokine blockade and metabolic reprogramming strategies with current immunotherapeutic modalities, it may be possible to reshape the immunosuppressive TME, reinvigorate effector immune responses, and enhance therapeutic efficacy across a broader range of cancer types.

**Table 2**, summarizes the shared metabolic consequences of CD38 activation (NAD^+^ depletion → Ca²^+^ flux → FAO) and highlights cell-type–specific features that distinguish Tregs, Bregs, MDSCs, TAMs, TANs, and regulatory NK cells. While the core pathway is conserved, each cell type exhibits unique signaling interactions, metabolic dependencies, and functional outputs that shape its immunosuppressive role in the TME.

**Table 2:** Comparative features of the CD38–NAD^+^–Ca²^+^–FAO metabolic axis across regulatory immune cell types.

Cell Type	CD38 Expression Pattern	Shared Metabolic Consequence (CD38→NAD^+^→Ca²^+^→FAO)	Functional Outcome	Cell-Type–Specific Features
Tregs	Upregulated in activated and tumor-infiltrating Tregs	NAD^+^ depletion impairs Ca²^+^ signaling and limits FAO-dependent mitochondrial fitness	Reduced suppressive capacity, FoxP3 instability under stress	Strong dependence on FAO for lineage stability; CD38 regulates IL-2 sensitivity and mTORC1 restraint (261)
Bregs	Elevated in IL-10^+^ Bregs and inflammatory conditions	Impaired SIRT-dependent mitochondrial metabolism when NAD^+^ is low	Reduced IL-10 production, diminished tolerogenic function	SIRT3 is uniquely sensitive to CD38/NAD^+^ levels; enhanced mitochondrial enzyme deacetylation required for Breg function (262),
MDSCs	High in potent suppressive monocytic and granulocytic subsets	Lower NAD^+^ disrupts Ca²^+^ flux and limits FAO needed for suppressive programs	Reduced ARG1, iNOS, ROS-dependent suppression	CD38 interacts with STAT3 and ER stress pathways to maintain suppressive phenotype; tumor-derived signals strongly induce CD38 (27)
TAMs	CD38^+^ TAMs enriched in M2-like, pro-tumoral states	FAO impairment shifts macrophage polarization away from suppressive M2-like state	Reduced IL-10, TGF-β, and tissue-repair programs	CD38 coordinates with adenosine/CD39/CD73 signaling; influences scavenger receptor expression and phagocytic phenotype (263)
TANs	CD38 upregulated in N2-like, pro-tumoral neutrophils	NAD^+^ decline and Ca²^+^ dysregulation impair mitochondrial reserve	Loss of suppressive or pro-angiogenic TAN function	FAO contributes to NETosis regulation; CD38 modulates cAMP/cADPR-driven Ca²^+^ mobilization uniquely in neutrophils (31)
NK-Regs	CD38 upregulated in exhausted/regulatory NK phenotypes	Reduced FAO and Ca²^+^ signaling weaken mitochondrial fitness	Diminished cytotoxicity, increased immunoregulatory cytokine secretion	CD38 regulates ADPR/cADPR-mediated Ca²^+^ flux more strongly than in other cell types; intersects with TIM-3 and TIGIT pathways in NK cells (264), (265)

### 6.2 The expression of CD38 on Tregs and functional implication in the TME

CD38 is constitutively expressed at varying levels on regulatory T cells (Tregs) and is frequently upregulated in response to inflammatory stimuli and within the TME. High CD38 expression identifies a functionally potent, highly suppressive Treg subset associated with enhanced immune tolerance in both autoimmune conditions and cancer.

Functionally, CD38^+^ regulatory T cells (Tregs) share key immunosuppressive mechanisms with CD38^+^ regulatory B cells (Bregs), notably through the production of anti-inflammatory cytokines such as IL-10 and TGF-β, and the activation of calcium-dependent signaling pathways (34, 120).

**Figure 3:**
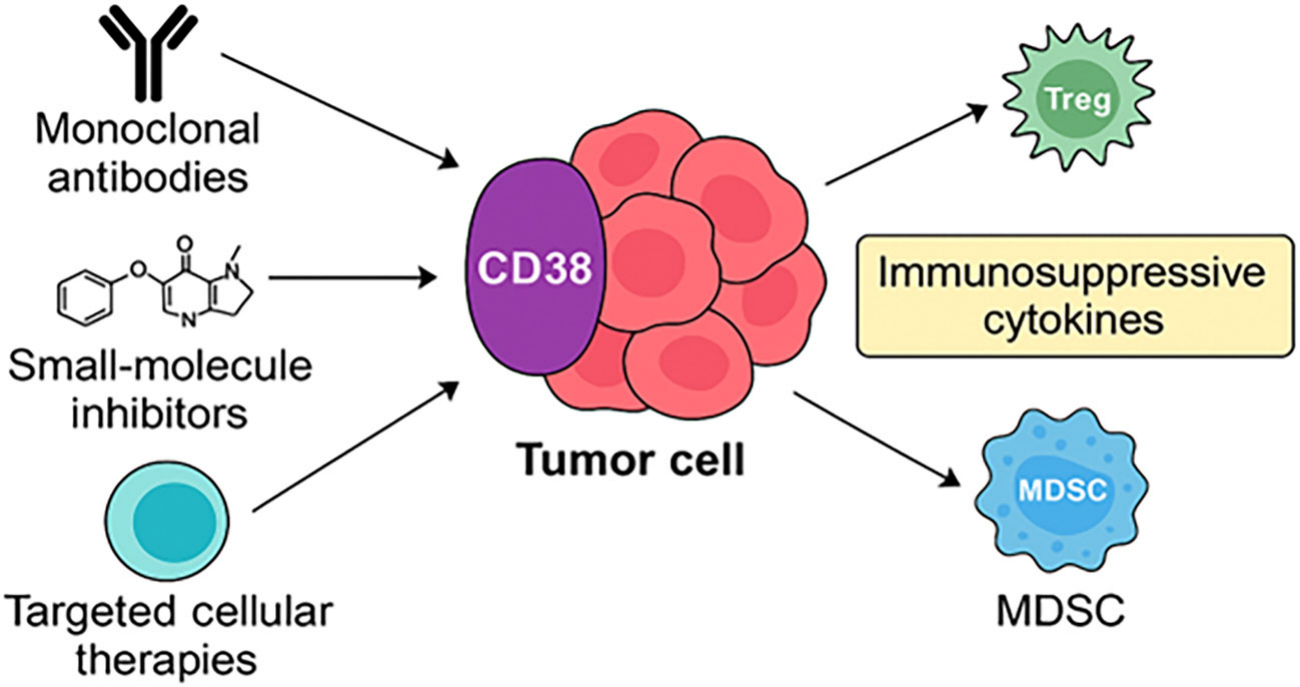
CD38–NAD^+^–Ca²^+^–FAO axis driving immunosuppressive functions in the tumor microenvironment. CD38, expressed on regulatory immune cells (Tregs, Bregs, MDSCs, TAMs, TANs, and regulatory NK cells), hydrolyzes NAD^+^ and NADP^+^ to generate second messengers such as cyclic ADP-ribose (cADPR) and NAADP. These metabolites mobilize intracellular Ca²^+^ stores, activating signaling pathways that promote fatty acid oxidation (FAO) and metabolic adaptation under hypoxic, nutrient-deprived conditions. NAD^+^ depletion further impairs glycolysis and oxidative phosphorylation in effector T cells, reinforcing immune suppression. Collectively, this axis sustains immunoregulatory cell survival and function, contributing to tumor immune evasion and resistance to immunotherapy.

The ecto-enzymatic activity of CD38 catalyzes the conversion of NAD^+^ to cyclic ADP-ribose (cADPR) and NAADP, potent second messengers that mobilize intracellular calcium (Ca²^+^) stores from the endoplasmic reticulum and lysosomes, respectively (217, 266–268). Elevated intracellular Ca²^+^ levels promote Treg activation, stabilize Foxp3 expression, and potentiate the secretion of suppressive cytokines. These calcium-mediated signals are crucial for maintaining Treg stability, metabolic adaptability, and survival, particularly within nutrient-deprived and hypoxic tumor microenvironments (TMEs) (269–271). Through these mechanisms, CD38^+^ Tregs are well-equipped to sustain immunosuppression under conditions that would otherwise compromise effector T cell function (**Figure 3**).

Importantly, CD38^+^ Tregs exhibit an enhanced capacity to suppress effector immune responses, particularly targeting CD8^+^ cytotoxic T lymphocytes (CTLs) and pro-inflammatory Th1 and Th17 subsets (42, 54). This heightened immunosuppressive activity contributes to immune evasion and tumor progression. Notably, increased infiltration or peripheral expansion of CD38^+^ Tregs has been associated with poor clinical outcomes across multiple solid tumor types, including melanoma, non-small cell lung cancer (NSCLC), and pancreatic ductal adenocarcinoma (PDAC) (272, 273).

Given their immunosuppressive potency, CD38^+^ regulatory T cells (Tregs) have emerged as a compelling therapeutic target in cancer immunotherapy due to their potent immunosuppressive activity and association with poor clinical outcomes across multiple tumor types (25, 274). Monoclonal antibodies targeting CD38, such as Daratumumab—currently FDA-approved for the treatment of multiple myeloma—have demonstrated potential beyond hematologic malignancies. In preclinical models and early translational studies, Daratumumab and similar agents have shown efficacy in depleting CD38^+^ Tregs (54, 275), thereby reducing immunosuppressive pressure in the TME and enhancing the therapeutic impact of immune checkpoint inhibitors (e.g., anti-PD-1, anti-CTLA-4) and adoptive T cell therapies including CAR-T and TIL therapies (54, 276).

However, CD38 expression is not limited to Tregs, and is also found on regulatory B cells (Bregs (120),, myeloid-derived suppressor cells (MDSCs), NK cells, and subsets of activated effector T and B cells. As such, broad depletion of CD38^+^ cells may lead to unintended immunological consequences, including impaired anti-tumor responses or immune dysregulation. To address this challenge, next-generation strategies are being developed to selectively modulate CD38^+^ Tregs without disrupting global immune homeostasis. These include antibody-drug conjugates (ADCs) with Treg-selective payloads (277), bispecific antibodies targeting CD38 and Treg-specific surface markers (e.g., CTLA-4 or TIGIT) (278, 279), engineered CAR-T cells designed to recognize and eliminate CD38^+^ Tregs specifically within the TME, and temporal or local delivery systems that minimize systemic exposure. Ultimately, integrating CD38-targeting approaches with immune checkpoint blockades or metabolic reprogramming may provide synergistic benefits, particularly in tumors with high CD38^+^ Treg infiltration and resistance to conventional immunotherapy.

### 6.3 The expression of CD38 on MDSCs and functional implication in the TME

CD38 is frequently upregulated on both monocytic (M-MDSCs) and granulocytic (PMN-MDSCs) subsets in various cancer types, particularly within the TME, where it plays a pivotal role in promoting immune suppression, tumor progression, and resistance to therapy. CD38^+^ MDSCs display a distinctly immunosuppressive phenotype, characterized by elevated expression of arginase-1 (ARG1) (27, 280), inducible nitric oxide synthase (iNOS) (281, 282), and reactive oxygen species (ROS) (9, 283, 284), which collectively impair CD8^+^ T cell proliferation and IFN-γ production (26, 27). As a multifunctional ectoenzyme, CD38 also governs NAD^+^ metabolism, leading to NAD^+^ depletion that disrupts mitochondrial function and shifts cellular energy utilization toward FAO and aerobic glycolysis (197, 241, 285). This metabolic reprogramming enables MDSCs to maintain functionality and survival in the hypoxic, nutrient-depleted conditions typical of the TME.

In addition, CD38 enzymatic activity results in the production of cyclic ADP-ribose (cADPR) and nicotinic acid adenine dinucleotide phosphate (NAADP) (217, 268), both of which mobilize intracellular calcium stores. The resulting rise in intracellular Ca²^+^ activates downstream signaling pathways—such as NF-κB and STAT3—that support MDSC survival, migration, and the secretion of immunosuppressive cytokines (286, 287), including IL-10 and TGF-β. CD38^+^ MDSCs contribute to the establishment of a tolerogenic and pro-tumorigenic microenvironment by directly suppressing T cell activation and cytotoxicity (28, 31), promoting regulatory T cell (Treg) expansion (54), and facilitating angiogenesis and metastatic progression via the secretion of vascular endothelial growth factor (VEGF), matrix metalloproteinases (MMPs), and other modulatory factors (31, 288, 289).

From a therapeutic perspective, targeting CD38 on MDSCs—using agents such as Daratumumab or Isatuximab—has shown efficacy in preclinical models of both solid tumors and hematologic malignancies. These strategies can mitigate MDSC-mediated suppression, restore antitumor T cell responses, and enhance the effectiveness of immune checkpoint blockade (37, 290). However, given that CD38 is also expressed on other immune cell subsets, including natural killer (NK) cells and activated T cells, selective targeting approaches will be essential to minimize off-target effects and preserve immune homeostasis.

### 6.4 The expression of CD38 on TAMs and functional implication in the TME

CD38 is increasingly recognized as a functionally important molecule expressed on TAMs within the TME. TAMs, particularly those polarized toward an M2-like immunosuppressive phenotype, frequently express high levels of CD38, which contributes to both their regulatory function and metabolic adaptation in cancer (29, 30, 291). Functional roles of CD38^+^ TAMs in the TME are immunosuppressive polarization and Cytokine Production. CD38^+^ TAMs display an M2-like phenotype, characterized by the production of IL-10, TGF-β, and arginase-1 (ARG1)—factors that suppress T cell responses and promote tumor immune evasion. CD38 expression is induced by TME-associated cues such as IL-4, IL-13, hypoxia, and tumor-derived exosomes, which favor M2 polarization (292, 293). These TAMs contribute to the recruitment and expansion of regulatory T cells (Tregs) and myeloid-derived suppressor cells (MDSCs), reinforcing immunosuppression (294, 295).

As an ectoenzyme, CD38 in TAM degrades extracellular NAD^+^, limiting substrate availability for NAD^+^-dependent enzymes like Sirtuins (30, 199, 296–298) and PARPs (36), thereby dampening pro-inflammatory macrophage functions and enhancing tolerance. This enzymatic activity shifts TAM metabolism toward FAO and supports survival in hypoxic, nutrient-depleted environments (197). CD38^+^ TAMs contribute to angiogenesis, extracellular matrix remodeling, and metastasis through the secretion of VEGF, MMPs, and other growth factors (29, 299). They also support tumor growth by enhancing stemness, invasion, and resistance to therapy in cancer cells through paracrine signaling. Through the production of cADPR and NAADP, CD38 modulates intracellular Ca²^+^ signaling, activating downstream transcription factors such as NF-κB, STAT3, and CREB, which sustain TAM activation, cytokine expression, and survival (29, 217, 300).

#### 6.4.1 Clinical implications and targeting CD38^+^ TAMs

High infiltration of CD38+ TAMs has been correlated with poor clinical outcomes in multiple solid tumors, including pancreatic ductal adenocarcinoma, colorectal cancer, and hepatocellular carcinoma (29, 30, 33, 301). Therapeutically, targeting CD38^+^ TAMs offer a promising strategy to reverse macrophage-mediated immunosuppression, bolster cytotoxic T cell responses, and enhance tumor sensitivity to immune checkpoint inhibitors (302–304). However, the pleiotropic expression of CD38 across various immune subsets-including regulatory T cells (Tregs), myeloid-derived suppressor cells (MDSCs), regulatory B cells (Bregs), and natural killer (NK) cells—poses a challenge for selective targeting (213, 305). Non-specific depletion may compromise immune homeostasis or dampen antitumor immunity. To address this, combination strategies are being explored. These include CD38 blockade in conjunction with macrophage reprogramming agents such as TLR agonists, CSF1R inhibitors, or CD40 agonists to skew TAMs toward an M1-like pro-inflammatory phenotype (139, 306–312);. Such approaches may synergistically dismantle immunosuppressive TME, overcome resistance to immunotherapy, and improve patient outcomes.

#### 6.4.2 The expression of CD38 on TANs and functional implication in the TME

Tumor-associated neutrophils (TANs) represent a highly plastic and heterogeneous population within the TME, capable of adopting either anti-tumor (N1) or pro-tumor (N2) phenotypes depending on contextual cues. Emerging evidence indicates that CD38 is upregulated on TANs, particularly those exhibiting pro-tumorigenic N2-like characteristics, and plays a critical role in shaping their immunosuppressive, inflammatory, and metabolic profiles (117, 313).

Functionally CD38^+^ TANs contribute to immune evasion by suppressing T cell proliferation, inhibiting CD8^+^ T cell cytotoxicity, and promoting regulatory T cell (Treg) expansion (31, 273). This is partly mediated by upregulated expressions of PD-L1, arginase-1 (ARG1), ROS, and immunosuppressive cytokines such as IL-10 and TGF-β. As with other myeloid subsets, CD38 acts as an NAD^+^-degrading ectoenzyme, thereby disrupting NAD^+^-dependent signaling pathways (e.g., SIRT1, PARPs) and altering neutrophil metabolic programming. CD38^+^ TANs show enhanced FAO and glycolytic activity, which supports their survival and function in hypoxic tumor regions (35, 191, 314, 315).

CD38^+^ TANs promote tumor growth and metastasis by releasing matrix metalloproteinases (e.g., MMP9), vascular endothelial growth factor (VEGF), and neutrophil extracellular traps (NETs) that remodel the extracellular matrix and facilitate invasion (47, 316–318). Through paracrine signaling, they support tumor cell stemness, angiogenesis, and resistance to therapy. The enzymatic products of CD38, cADPR and NAADP, mobilize intracellular Ca²^+^ stores and activate transcription factors such as NF-κB, STAT3, and CREB, which further enhance TAN activation, cytokine secretion, and survival (217, 319–321).

Clinically, high infiltration of CD38^+^ TANs has been associated with poor prognosis and resistance to immunotherapy in lung cancer (272, 322), gastric cancer (323–325), and head & neck squamous cell carcinoma (318, 326). Targeting CD38^+^ TANs, either alone or in combination with checkpoint inhibitors or NET inhibitors, is under preclinical investigation as a strategy to reduce neutrophil-mediated immunosuppression and enhance anti-tumor immune responses.

#### 6.4.3 The expression of CD38 on regulatory NK cells and functional implication in the TME

Regulatory natural killer (NK) cells, often identified by the phenotype CD56^bright CD16^-^ NKG2D^+^ TIGIT^+^ CD38^+^, represent a subset of NK cells with immunosuppressive properties. These cells express high levels of CD38, a multifunctional ectoenzyme involved in NAD^+^ metabolism, calcium signaling, and immune regulation (196, 327).

In the TME, CD38 expression on regulatory NK cells is functionally significant for several reasons: 1) Immune Suppression: CD38^+^ regulatory NK cells produce immunosuppressive cytokines, notably IL-10, which inhibit the activation and cytotoxic function of effector CD8^+^ T cells and conventional NK cells. This cytokine milieu contributes to an immune-tolerant TME that supports tumor progression (173, 328, 329). 2) Metabolic Modulation: CD38 enzymatic activity leads to NAD^+^ depletion and generation of metabolites such as cyclic ADP-ribose, which modulate intracellular calcium signaling pathways. This metabolic rewiring can suppress NK cell cytotoxicity and promote regulatory phenotypes, further dampening anti-tumor immune responses (330–332). 3) Regulation of Immune Crosstalk: CD38 facilitates interactions between regulatory NK cells and other immunosuppressive populations within the TME, such as Tregs, MDSCs, and TAMs. These interactions amplify the immunosuppressive network, reinforcing immune escape mechanisms. 4) Resistance to Immunotherapy: High CD38 expression on regulatory NK cells correlates with poor responses to immune checkpoint blockade (ICB) therapies (e.g., anti-PD-1, anti-CTLA-4), as these cells contribute to persistent immune suppression despite therapy (322, 333).

Therapeutically targeting CD38 on regulatory natural killer (NK) cells using monoclonal antibodies, such as Daratumumab and Isatuximab, or small molecule inhibitors, represents a promising approach to modulate the immunosuppressive landscape within the TME (327, 334). By specifically depleting or inhibiting these CD38-expressing regulatory NK cells, it is possible to disrupt their suppressive cytokine production and metabolic pathways that contribute to immune evasion. This disruption can restore the cytotoxic function of conventional NK cells and effector T cells, thereby enhancing their ability to recognize and eliminate tumor cells (31, 327, 335, 336). Furthermore, CD38-targeted therapies may synergize with current immune checkpoint blockade treatments by reversing resistance mechanisms linked to the immunosuppressive activity of regulatory NK cells (40, 337, 338). Ultimately, these interventions aim to recondition the TME from an immunosuppressive to an immunostimulatory state, improving anti-tumor immunity and clinical outcomes in patients with solid tumors.

## 7 Regulating the regulators via targeting CD38

Targeting CD38 to disarm immune suppression in TME. The TME is characterized by a complex network of immunosuppressive cell populations-including Tregs, Bregs, MDSCs, TAMs, and TANs that collectively inhibit effective anti-tumor immune responses. A shared feature among many of these immunosuppressive subsets is the expression of CD38, a multifunctional ectoenzyme and signaling molecule that plays a central role in shaping the suppressive landscape of the TME.

### 7.1 CD38 as a master regulator of immune suppression

**Figure 4:**
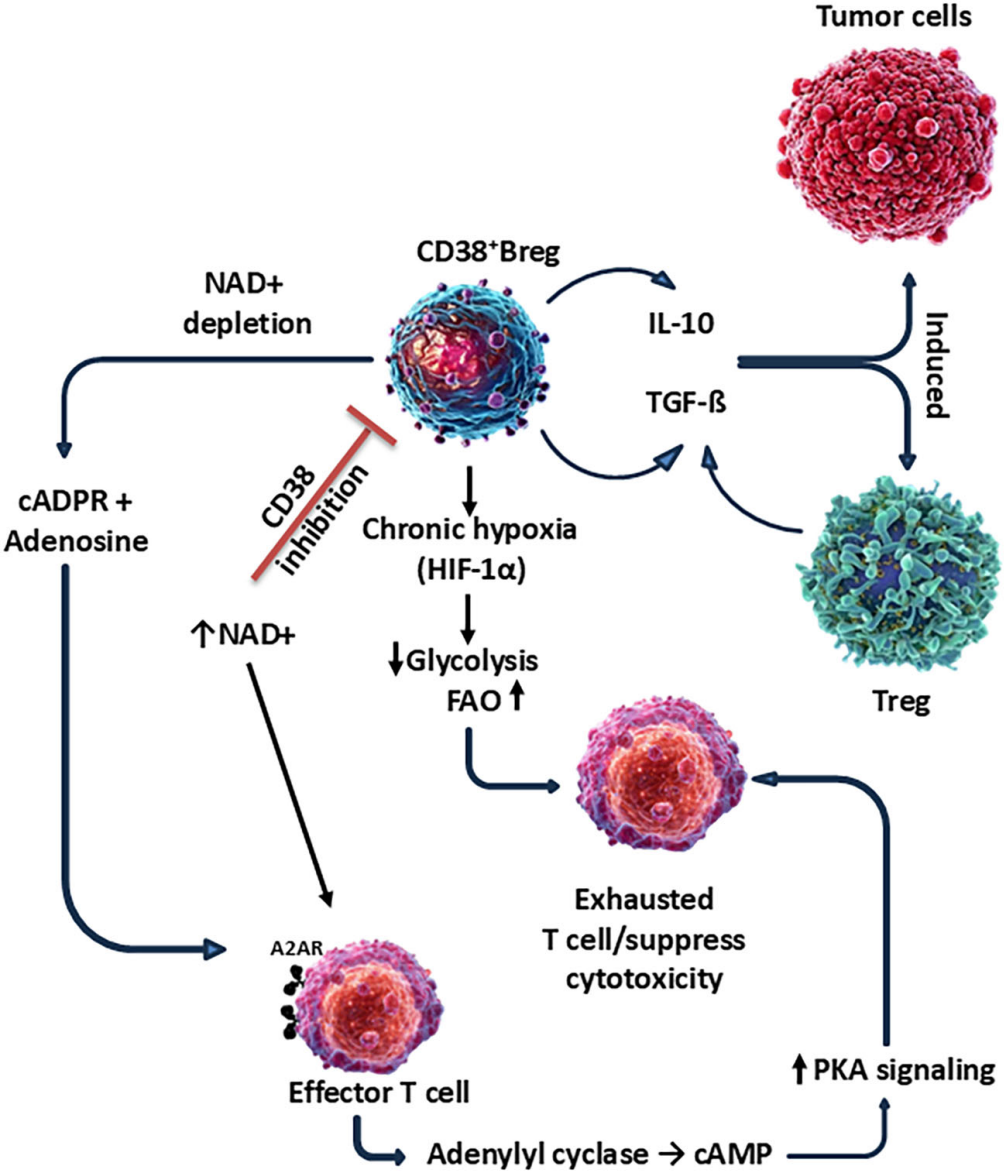
Therapeutic strategies targeting CD38 and associated immunosuppressive networks. CD38 is highly expressed on tumor cells and contributes to immunosuppression through NAD+ metabolism and signaling pathways. Current therapeutic approaches include monoclonal antibodies (e.g., daratumumab, isatuximab), small-molecule inhibitors that block CD38 enzymatic activity, and targeted cellular therapies such as CAR-T cells. CD38-driven immunosuppressive networks involve regulatory T cells (Tregs), myeloid-derived suppressor cells (MDSCs), and immunosuppressive cytokines (e.g., IL-10, TGF-β), which collectively dampen anti-tumor immunity.

CD38 is involved in both enzymatic and receptor-mediated functions that converge to enhance the survival, function, and metabolic adaptation of regulatory immune cells. Its key roles include NAD^+^ hydrolysis (191, 213, 305), which deprives cells of NAD^+^ required for metabolic fitness and DNA repair, Generation of second messengers (cADPR, NAADP), leading to Ca²^+^ mobilization and activation of transcription factors like STAT3, NF-κB, NFAT (35, 236, 241, 314), and CREB and Promotion of FAO and glycolysis, supporting immunosuppressive cell survival in nutrient-depleted, hypoxic environments (230), (**Figure 4**).

### 7.2 Targeting CD38 to reverse immune suppression

Elevated levels of CD38^+^ immunosuppressive cells correlate with poor prognosis, treatment resistance, and shorter survival across multiple cancers (e.g., NSCLC, pancreatic cancer, HCC, and melanoma). Early-phase clinical trials are investigating CD38-targeted therapies in solid tumors and their potential to sensitize tumors to immunotherapy. Therapeutic strategies aimed at targeting CD38 offer the potential to simultaneously deplete or reprogram multiple immunosuppressive cell populations in the TME (40, 42, 190). These approaches include monoclonal Antibodies (mAbs) against CD38, e.g. Daratumumab, Isatuximab, and MOR202-originally developed for multiple myeloma—can deplete CD38^+^ regulatory cells via antibody-dependent cellular cytotoxicity (ADCC) (37, 339), complement-dependent cytotoxicity (CDC) (340), or direct apoptosis (275). Depletion of CD38^+^ Tregs, MDSCs, TAMs, and Bregs may reduce immune suppression and restore cytotoxic T lymphocyte (CTL) function. Anti-CD38 with anti-PD-1/PD-L1 or anti-CTLA-4 (341) may overcome resistance by lifting the immunosuppressive barrier. Combining CD38 blockade with metabolic inhibitors (FAO or IDO inhibitors) may reprogram the TME to favor effector immune cell activity. CD38 targeting drug along with TAM reprogramming agents (CSF1R inhibitors (342), TLR agonists (37, 343), or CD40 agonists) (344) can synergize with CD38 blockade to re-educate TAMs toward a pro-inflammatory (M1-like) phenotype. Given CD38’s expression on both regulatory and effector immune cells (e.g., NK cells, activated T cells), selective targeting is essential to avoid compromising anti-tumor immunity. Bispecific antibodies (345, 346), antibody-drug conjugates (ADCs) (347), or nanoparticle-based delivery (348, 349) may improve specificity and reduce systemic toxicity.

In addition to antibody-based therapies, several small molecule inhibitors of CD38 enzymatic activity are under investigation. These include NAD analogs (e.g., Ara-F-NAD, Ara-F-NMN, Carba-NAD, Pseudocarba-NAD), Flavonoids (e.g., Apigenin, Luteolinidin, Kuromanin, Rhein/K-rhein), 4-Aminoquinolines (e.g., 78c, 1ah, 1ai) (36, 199, 298, 350). Our novel observation highlights that a single molecular target—CD38—can be therapeutically leveraged to eliminate both Bregs and Tregs. This strategy is especially promising in CD38-high, immune-suppressive hematologic malignancies such as CLL and MM, and may be equally effective in other solid tumors or disease states where regulatory cells drive immune evasion. “Beyond cancer, CD38 plays a significant role in autoimmune and inflammatory disorders, where its enzymatic activity influences NAD^+^ metabolism and immune regulation. Elevated CD38 expression has been linked to pathogenic T-cell subsets in systemic lupus erythematosus and rheumatoid arthritis, as well as B-cell hyperactivity in autoimmune diabetes. These observations underscore CD38’s broader relevance as a metabolic checkpoint in immune homeostasis. However, the mechanistic parallels between autoimmunity and tumor immunology remain incompletely defined, highlighting an opportunity for cross-disciplinary insights into CD38-targeted interventions”.

Moreover, CD38 targeting holds translational promise in a range of immune-mediated and autoimmune diseases where immune regulatory cells (more precisely Bregs and Tregs) are pathologically expanded, including Systemic Lupus Erythematosus (SLE) (24, 351–354), Rheumatoid Arthritis (RA) (355), Celiac Disease (356), Sjögren’s Syndrome (357), Polymyalgia Rheumatica (PMR) (358), Multiple Sclerosis (MS) (359), Ankylosing Spondylitis (AS) (360), Type 1 Diabetes (361) Alopecia Areata (AA) (362) and Vasculitis. Additionally, CD38 has been recently implicated in cellular senescence, mitochondrial biogenesis, and mitochondrial trafficking, particularly in the context of aging and age-related malignancies, further underscoring its therapeutic value.

## 8 Conclusion

Targeting CD38 offers a unique opportunity to “regulate the regulators” within the TME. This review summarizes how CD38-driven NAD^+^ depletion, Ca²^+^ signaling, and FAO collectively sustain regulatory networks and how targeting CD38 offers a unique opportunity to dismantle these interconnected suppressive circuits. By disrupting the metabolic, enzymatic, and signaling functions that sustain Tregs, Bregs, MDSCs, TAMs, and TANs, CD38 blockade could unlock the full potential of immunotherapies and improve clinical outcomes in cancer patients. “There is strong consensus that CD38 functions as a multifunctional ectoenzyme and receptor, influencing NAD^+^ metabolism, Ca²^+^ signaling, and immunosuppressive programming in Tregs, Bregs, MDSCs, TAMs, and TANs. Its role in shaping the TME and contributing to resistance against immune checkpoint blockade (ICB) is well supported. However, several uncertainties remain: 1) The precise role of CD38 in iNKT cell fate is incompletely understood (330), with conflicting evidence on whether CD38 promotes activation or exhaustion. 2) The selectivity challenge of CD38-targeted therapies—avoiding collateral depletion of effector immune cells while eliminating suppressive subsets—requires further investigation and innovative strategies and 3) Whether CD38 inhibition alone is sufficient to reverse ICB resistance remains unclear; emerging data suggest that combination approaches (e.g., CD38 blockade with checkpoint or FAO inhibitors) may be necessary for durable responses (363). Addressing these gaps will be critical for translating CD38 biology into effective and safe immunotherapeutic strategies”.

Rational combination approaches and selective targeting strategies will be key to maximizing efficacy while preserving beneficial immune functions. in summary, CD38 represents a versatile and promising target for both molecular therapy and immunotherapy. Its broad expression on immune suppressive regulatory cells and role in cancer progression and immune dysfunction make it a compelling candidate for future clinical applications.
